# Sulphide of Calcium in the Treatment of Supperating Buboes

**Published:** 1880-06-20

**Authors:** Fessenden N. Otis


### SULPHIDE OF CALCIUM IN THE TREATMEMT OF SUPPURATING BUBOES.

BY FESSENDEN N. OTIS, M. D.

My attention was first called to the value of the sulphide of calcium in arresting processes of suppuration through an article in the Lancet of February 21, 1874, by Sidney Ringer, M.D. Dr. Ringer claimed that, when the product of suppuration in scrofulous sores was thin and ichorous, the administration of small doses of the sulphide of potassium or of calcium promptly changed the purulent fluid to one of a more healthy character, and that the healing of the sore was promoted. He also claimed that the formation of boils and abscesses was prevented by a timely administration of small doses of the sulphides, and that, when suppuration had already occurred in such cases, the suppurative process was quickly arrested through the influenee of these remedies. Opportunity for a practical test of these claims soon occurred, and resulted in my own personal conviction of their entire correctness, and I have now for the last five years habitually prescribed the sulphide of calcium in cases of threatened suppuration in phlegmonous swelling from various causes, and, as a rule, with very gratifying results. The manner of its use was practically the same as advised by Dr. Ringer, viz: 1-12 grain of the sulphide of calcium every two hours, or 1-20 every hour, during the day and up to the time of retiring. Especially have I found small doses of the sulphide of calcium useful in arresting the progress of furuncular swellings and abscesses, and in preventing their occurrence when threatening. On the other hand, I have repeatedly tested the influence of this drug upon the suppurative processes in mucous membranes, as in gonorrhea, gleet, leucorrhea, etc., without being able to discover that it influenced or modified the suppurative process in such cases in the least degree.

Among the cases in my private practice where prompt arrest of suppuration was quickly followed by absorption of pus already formed and resolution of tumor, and apparently from the use of the sulphide of calcium, were several inguinal buboes associated with chancroid. The simple fact that resolution occurred in these cases were (in accordance with the popular teaching) accepted as proof that the buboes were of sympathetic and not of chancroidal origin.

Authorities have long taught that, once the virus from a chancroid has been carried along a lymphatic vessel and deposited in the adjacent lymphatic gland, inflammation is at once set up in the substance of the gland. This, it is claimed, goes steadily on in spite of all and any treatment until an abscess is formed. This must, sooner or later, through advance of the suppurative agency or by surgical interference, result in an open ulcer, the pus of which will possess the same vicious character as the chancroid from which it was derived. This variety of bubo is known as the virulent or chancroidal bubo. The suppuration of such buboes has been considered inevitable, and all buboes not pursuing this course have been set down as not of true chancroidal but of simple or sympathetic origin. Inflammatory lymphatic enlargements associated with chancroid are very naturally dreaded as most likely to prove by results to be of chancroidal origin, and usually, after a few feeble attempts at treatment with a view to their resolution, glands affected are encouraged to suppurate, and prompt incision and evacuation of pus are advised as soon as the slightest true fluctuation is recognized. If suppuration is indeed inevitable, undoubtedly it is wise to encourage it, to evacuate the virulent product at the earliest moment, and thus afford access for efficient treatment for the destruction of this new-formed chancroid. For this reason I had been an earnest advocate for early incision into suppurating buboes associated with chancroid. My experience in the few cases above alluded to, however, made me incline to the belief that a thorough and extended trial of the calcium sulphide in cases of inflammatory buboes associated with chancroid might give such results as to make its use imperative in every such case.

In order to gain further light on this important matter a systematic use of the calcium sulphide was made, in my service at Charity Hospital, in eighteen consecutive cases of inflammatory bubo occurring with, or as the immediate result of, well-pronounced chancroid. All the facts considered of importance were noted by myself and under my direction by Dr. Johnson, my house surgeon, and are truly confirmatory.

Thus it will be seen that, out of eighteen cases of inflammatory bubo presenting the rational evidences of chancroidal origin, and treated systematically by the use of small doses of the sulphide of calcium, resolution occurred in fifteen, and that in only three cases was incision ultimately required.

If we apply to these cases the usual rule that chancroid buboes always eventuate in chancroidal abscesses, always suppurate and require evacuation by natural means or surgical procedure, then we must hold that only three out of fifteen cases of inflammatory buboes associated with chancroid were the result of transference of the suppurative process from the chancroid to the adjacent lymphatic gland. It is just possible, however, that the influence of the sulphide of calcium may, in arresting suppuration, extend to the true chancroidal bubo. The apparent successful use of this drug in the series of cases herewith presented at least suggests and invites a trial of its efficacy in all instances of threatened glandular suppuration, whether associated with chancroid or of purely sympathetic origin.—*N. Y. Med. Rec.*

