# Correction

**Published:** 1880-08-20

**Authors:** 


### EDITORIAL AND MISCELLANEOUS.

☞ Subscribers in arrears are requested to remit at once. We desire our friends not to send money to third parties for us, if it can be avoided. The mail is the best and safest medium and gives least trouble to all parties.

*Correction.*—The article, in our June number, on "*Sulphide of Calcium in the Treatment of Suppurating Buboes,*" credited to the New York Medical Record, should have been credited to the New York Medical Journal, written by F. N. Otis, M.D.

*The Fourth Annual Meeting* of the Dermatological Association will be held at the Ocean House, Newport, R. I., on the 31st of August, and the 1st and 2d of September, 1880. Many interesting papers will be presented.

*Monument to Claude Bernard.*—Dr. E. C Seguin, of New York, has been appointed by the Paris Committee to solicit subscriptions in behalf of a monument to the late Prof. Claude Bernard, the illustrious physiologist. He should be addressed at 41 W. Twentieth street, New York

*The Southern Medical College.*—The outlook for this new and rapidly rising Institution is very encouraging for the next session, which opens on the 13th of October next, as may be seen by advertisement in this Journal.

Facilities of a superior kind, not mentioned in the catalogue, will be furnished the students of this Institution. In fact it is believed that few schools can be found in the United States that can furnish superior advantages to those provided by the Southern Medical College.

*Bogus Diplomas.*—Recent disclosures show that the bogus diploma factory—at the head of which is one Prof. John Buchanan, of Philadelphia—has been running a brisk business for many years. The names of thousands of parties are published who hold these spurious diplomas, amongst whom we note some well known practitioners of Georgia. At present we publish no name, but may do so hereafter.

Legal proceedings have been instituted against the so-called College, and all those who hold and have been practicing under the bogus diplomas are liable to prosecution and heavy penalties, and it is the duty of our State Medical Boards to prosecute all such parties.

*Wm. R. Warner & Co.*—The most beautiful pocket drug case we have yet seen is one presented to us by that staunch and liberal establishment, the House of Wm. R. Warner & Co., Philadelphia.

The case is a beautiful red morocco of convenient size for the pocket, and contains ten two drachm phials filled with parvules of various kinds. These parvules are, as the name implies, very small and contain fractional doses of choice medicines.

We have found the parvules very convenient in practice, as we can graduate the dose to any required quantity without the trouble of compounding or mixing, which, to a physician in active practice, especially in the village or country, is a matter of no small importance. It saves time, trouble and annoyance, and at the same time assures accuracy of dose and purity of the medicine, as the drugs emanating from the House of Wm. R. Warner & Co. have an established reputation for integrity and reliability.

